# Passive immunization against highly pathogenic Avian Influenza Virus (AIV) strain H7N3 with antiserum generated from viral polypeptides protect poultry birds from lethal viral infection

**DOI:** 10.1186/1743-422X-5-144

**Published:** 2008-11-28

**Authors:** Mirza Imran Shahzad, Khalid Naeem, Muhammad Mukhtar, Azra Khanum

**Affiliations:** 1 Department of Biochemistry, Pir Mehr Ali Shah Arid Agriculture University, Murree Rawalpindi-46300, Pakistan; 2 National Reference Laboratory for Poultry Diseases (NRLPD), Animal Sciences Institute, National Agricultural Research Center (NARC), Islamabad, Pakistan

## Abstract

Our studies were aimed at developing a vaccination strategy that could provide protection against highly pathogenic avian influenza virus (AIV), H7N3 or its variants outbreaks. A purified viral stock of highly pathogenic H7N3 isolate was lysed to isolate viral proteins by electrophresing on 12% sodium dodecyl sulfate polyacrylamide gel electrophoresis (SDS-PAGE), followed by their elution from gel through trituration in phosphate buffered saline (PBS). Overall, five isolated viral polypeptides/proteins upon characterization were used to prepare hyperimmune monovalent serum against respective polypeptides independently and a mixture of all five in poultry birds, and specificity confirmation of each antiserum through dot blot and Western blotting. Antiserum generated from various group birds was pooled and evaluated in 2-week old broiler chicken, for its protection against viral challenge. To evaluate *in-vivo *protection of each antiserum against viral challenges, six groups of 2-week old broiler chicken were injected with antiserum and a seventh control group received normal saline. Each group was exposed to purified highly pathogenic AIV H7N3 strain at a dose 10^5 ^embryo lethal dose (ELD_50_). We observed that nucleoprotein (NP) antiserum significantly protected birds from viral infection induced morbidity, mortality and lowered viral shedding compared with antiserum from individual viral proteins or mixed polypeptides/proteins inclusive of NP component. The capability of individual viral polypeptide specific antisera to protect against viral challenges in decreasing order was nucleoprotein (NP) > hemagglutinin (HA) > neuraminidase (NA) > viral proteins mix > viral polymerase (PM) > non-structural proteins (NS). Our data provide proof of concept for potential utilization of passive immunization in protecting poultry industry during infection outbreaks. Furthermore conserved nature of avian NP makes it an ideal candidate to produce antiserum protective against viral infection.

## Background

Avian influenza virus (AIV) besides reducing commercial production of poultry is also a causative agent for influenza among humans by cross-species infections [1]. The viral genome encodes 10 proteins and among these two surface proteins haemagglutinin and neuraminidase have main importance in viral classification [2]. AIV grouping is based on antigenic variations in haemagglutinin (H1 – H16) and neuraminidase (N1 – N9) proteins and each strain of virus is named based on respective H and N antigenicity [3]. According to virulence pattern in poultry, the AIV is mainly classified into two major groups: highly pathogenic avian influenza (HPAI) and low pathogenic avian influenza (LPAI). The HPAI strains are highly virulent and associated with bird mortality approaching 100%, whereas LPAI viruses manifest mild symptoms like decreased egg production and scruffy feathers. Throughout the world majority of avian influenza epidemics are due to HPAI viruses showing H5 and H7 antigenicity [4,5]. In Pakistan, low pathogenic H9N2 along-with high pathogenic H7N3 and H5N1 are the most predominant AIV strains and several outbreaks over the past decades are ascribed to these particular strains [6-8].

Avian influenza (AI) has emerged as a disease with significant potential to disrupt commercial poultry production, resulting in heavy losses to poultry farmers in several parts of the world. Due to fastidious viral genome, conventional antivirals against AIV are unable to control the infection and very few effective vaccines are available. Moreover, geographic strain variations have made it difficult to implement universal avian influenza vaccine strategy. As such, there has been an urgent need to develop broad spectrum antivirals against AIV or vaccines capable of coping with viral genomic changes. One of the most plausible options to control AI is development of regional immunization programs against the serotype involved in an outbreak. However, as the immunization has to be carried out prior to disease for establishing therapeutic levels of antibodies against the infection, in case of its sudden outbreak such control measures are not possible. Passive immunization has emerged as an effective therapeutic tool in the face of an outbreak; however its effectiveness in the case of AIV has not yet been investigated. During past decade, AIV, H7 serotype has caused high poultry birds mortality in different countries including Pakistan [6]. The whole virus killed AIV vaccines used against H7 has been found to be effective in reducing the clinical conditions of the birds upon subsequent field challenge [2]. However, practically it is always difficult to make use of any kind of killed vaccines during the outbreaks due to very short incubation period associated with highly pathogenic AI infection. Keeping this in view, the present study was designed to compare various viral proteins for their potentials as a vaccine candidate. According to our data nucleoprotein (NP) antiserum significantly protected birds from viral infection induced morbidity/mortality and lowered viral shedding compared with antiserum from other viral proteins like hemagglutinin (HA) neuraminidase (NA), viral polypeptides mix, non structural protein and viral polymerase enzyme. This proof of concept study provides initial data to rely on utilization of individual viral protein for passive immunization programs.

## Results

**Figure 1:**
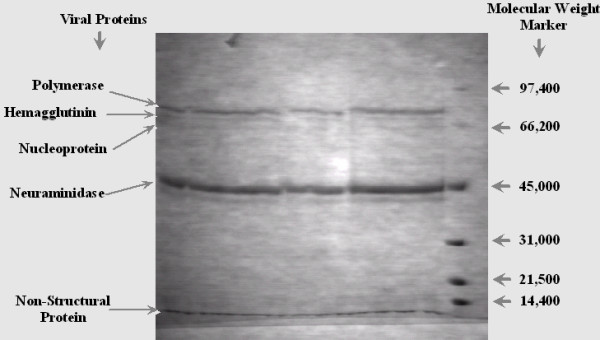
**SDS-PAGE analysis of avian influenza Virus strain H7N3 proteins**. Five major viral proteins are marked on gel corresponding to their molecular weight ascertained through protein molecular weight marker.

**Figure 2:**
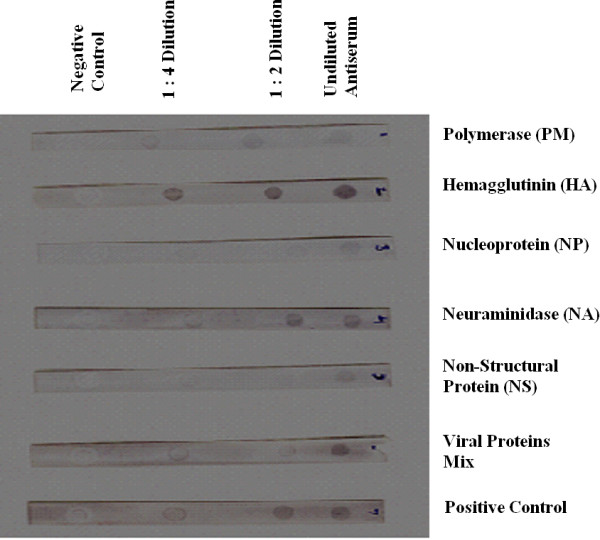
**Dot-ELISA**. confirms the antiserum specificity against respective polypetide.

Our initial work on SDS-PAGE analysis of H7N3 viral lysate showed five major viral proteins: high molecular weight polymerase (PM), hemagglutinin (HA), nucleoprotein (NP), neuraminidase (NA) and non-structural protein (NS) as shown in Figure 1. These polypetides were further concentrated and subjected to electrophoresis on SDS-PAGE. Five obvious bands of AIV viral polypetides were cut from the gel, triturated and diluted with 1.0 ml of normal saline. This follows generation of polypeptide specific antibodies against each polypeptide and also a mixture of all was used to generate antisera. The specificity of each polypeptide antiserum was confirmed by Dot-ELISA. Intriguingly, the viral peptides mix antisera detected H7N3 viral particles at 1:4 dilution (Figure 2).

All the birds used in this study were confirmed negative against AIV H7N3 antibodies by HI test. Passive immunization with individual polypeptide/protein specific antisera followed challenge with highly pathogenic AIV, H7N3. After 48 hours birds immunized with antisera and non-immunized control group were challenged with 0.2 ml of H7N3 viral strain A/Chicken/Pakistan/Murree/NARC/69/04 (H7N3). Birds' morbidity, mortality and cloacal shedding were observed over a time period of two-week. Four out of the six vaccinated group showed protection from lethal viral challenge whereas negative control group showed highest level of mortality, morbidity and cloacal shedding. The level of protection in the four groups varied and nucleoprotein antiserum vaccinated group birds showed highest protection revealed by least mortality, and low viral shedding (60%). The birds passively immunized with polymerase and non-structural protein antiserum showed no protection at all. Upon viral challenge, seven out of ten birds died in polymerase and non-structural protein antiserum vaccinated groups, whereas eight in non-vaccinated control group (Table 1). This trend continued and on day 4^th ^all the birds in PM, NS and control (normal saline group) were dead. Mortality was associated with extensive morbidity in polypeptides groups showing less protection. One of the groups was vaccinated with antiserum generated from a mixture of all five peptides (viral polypeptides mix group). It was intriguing to note that on day 4^th ^two birds died in this group without any further mortality thus showing 80% protection. No mortality (100% protection) was observed in birds pre-vaccinated with hemagglutinin, nucleoprotein, and neuraminidase antisera. However, morbidity and viral shedding revealed 80–100% birds infected in HA vaccinated group, 20 – 60% in NP and 80–100% in NA groups (Table 1).

**Table 1:** Protection of vaccinated poultry birds against highly pathogenic AIV H7N3 strain

**Groups**	**Antisera against viral protein(s)**	**Post-challenge mortality at different days**				**Post-challenge morbidity at different days**				**Post-challenge cloacal shedding at different days**			
		**2**	**4**	**7**	**14**	**2**	**4**	**7**	**14**	**2**	**4**	**7**	**14**

1	**Viral polymerase**	7/10	10/10	--	--	3/10	--	--	--	3/10	--	--	--

2	**Hemagglutinin**	0/10	0/10	0/10	0/10	4/10	8/10	8/10	0/10	10/10	10/10	8/10	2/10

3	**Nucleoprotein**	0/10	0/10	0/10	0/10	2/10	6/10	5/10	0/10	6/10	6/10	5/10	0/10

4	**Neuraminidase**	0/10	0/10	0/10	0/10	9/10	10/10	8/10	0/10	10/10	10/10	8/10	3/10

5	**Non-structural protiens**	7/10	10/10	--	--	3/10	--	--	--	3/10	--	--	--

6	**Viral polypeptides mixed**	0/10	2/10	0/8	0/8	10/10	8/8	7/8	0/8	10/10	8/8	7/8	2/8

7	**Normal saline**	8/10	10/10	--	--	2/10	--	--	--	2/10	--	--	--

Morbidity describes disease condition and prevalence of various symptoms associated with viral infection in birds. In case of bird flu outbreak, the infected birds manifest quite distinctive symptoms like ruffled feathers, excessive thirst, areas of diffuse hemorrhage between the hocks and feet, edema surrounding the eyes, watery green diarrhea progressing to white and several others. Mortality in the control (non-vaccinated) and two of the viral peptides (PM, NS) antisera manifesting least protection (0%) was associated with several disease symptoms an indicator for high morbidity (100%). In comparing the data of all protective antisera groups, the level of morbidity was higher in viral polypeptides and neuraminidase groups (100%) followed by hemagglutinin (80%) on day 4^th^. The nucleoprotein antiserum immunized group showed the least morbidity (maximum 60%) at day 4 along-with no mortality (0%) and lowest level of cloacal shedding makes it a potential candidature for poultry vaccine against H7N3 especially through passive immunization route.

In vaccinated groups challenged with lethal AIV, NP groups showed least cloacal shedding of virus among all the groups. All other vaccinated and non-vaccinated control manifested cloacal shedding of virus. These data are quite interesting and will help us in designing future vaccine for AIV in poultry.

## Discussion

Infections associated with AIV are threatening economy of several countries throughout the World. Particularly in South-East Asia viral infection has inflicted major losses to poor poultry farm holders as well as it poses a threat of cross-species infection among humans. AIV is a member of Type A group viruses and compared with its counterparts Type B and C has broad host range capable of causing infections in several birds and mammals. One of the major threats of AIV has been its capability to cross-species jumping i.e. from birds to humans [9].

According to a report from the International Federation for Animal Health (IFAH) vaccination strategies for controlling AIV infection in birds is one of the major viable options compared with other control measures [10]. Several vaccine strategies including production of vaccine from virus like particles are on horizon [11,12]. Killed vaccines have also been considered to control viral pandemic in flocks in-spite of its limitation in surveillance programs involving differentiation of infected from vaccinated animals (DIVA) test [2] particularly if killed vaccines are being used. For differentiating vaccinated birds from the naturally infected ones DIVA test strategy relies on detecting antibodies against N-type only found in infected birds and not against serotype of vaccine strain, besides general monitoring strategy of unvaccinated sentinels.

Passive immunization with antiserum generated from viral polypeptides antigenic determinants has shown significant protection in mammals [13,14] and also in birds [15]. We employed a passive immunization strategy by utilizing various proteins of AIV to ascertain which one of these could be comparatively a better candidate for the generation of antisera to be used for passive immunization. The viral polypeptides used in this study were from a highly pathogenic avian influenza virus serotype H7N3 that has been previously reported in Pakistan and several other parts of the world [6,7,16]. Our proof of concept studies reveal that it is possible to develop passive immunization strategies against AIV subtype by using viral proteins and among the five viral proteins (hemagglutinin, neuraminidase, nucleoproteins, non-structural protein, polymerase, and a mixture of all these) nucleoprotein generated antiserum provided better protection in birds upon challenge with highly pathogenic avian influenza virus.

Four out of six vaccines have given protection in decreasing order NP>HA>NA>viral polypeptides mix. In case of HA, NA and viral polypeptides mix, the level of infection increased from day 0 to day 4 and then it decreased till the end of experiment i.e. day 14. NP antiserum besides providing 100% protection also boosted chick's immunity manifested as sustained resistance against infection (low level of morbidity and viral shedding) as compared to other vaccine groups. These data suggest that passively transfused anti-NP antibodies have a better antiviral neutralizing effect and overall protection from AIV. Overall, a better protection provided during days 7–14 is due to immune regulation.

Considering the situation of developing nations like Pakistan passive immunization strategy will be economical and targeted. Avian Influenza is capable of changing antigenic determinants that leads to inefficacy of vaccines. A locally produced economical vaccine will provide effective and long lasting solution to this pandemic especially the non-variant parts (nucleoproteins) that hold the promising future of AIV vaccines.

## Materials and methods

Prior to beginning this study the protocol was reviewed and approved by the animal biosafety committee of the Pir Mehr Ali Shah Arid Agriculture University Rawalpindi, and all the viral challenges and preparations were conducted at the biosecure facilities of the National Reference Laboratory for Poultry Diseases (NRLPD) at the Animal Sciences Institute, National Agriculture Research Center (NARC), Islamabad, Pakistan.

### Viral stocks

A previously isolated highly pathogenic AIV serotype H7N3 A/Chicken/Pakistan/Murree/NARC/69/04 (H7N3) [17] was obtained from the repository of the NRLPD at Animal Sciences Institute, National Agricultural Research Center (NARC), Islamabad. The viral stock was reactivated in the allantoic cavity of embryonated hen's eggs as described previously [18]. Agar gel precipitation test was used to confirm the presence of AIV in the allantoic fluid [19] and HA test was performed to calculate the viral titer, whereas embryo lethal dose 50 (ELD_50_) titer of the fresh viral stock was determined by classical Reed and Muench [20] methodology. In brief, this involves 10 fold serial dilutions of stock virus in normal saline (10^1 ^to 10^12^) followed by injecting 0.2 ml of each dilution into the chorioallantoic region of embryonated eggs. The mortality of eggs is recorded and ELD_50 _calculated as described previously[20].

### Preparation of viral polypeptides and production of monovalent hyperimmune antisera

Purified fresh stock of H7N3 AIV was lysed with 4% Triton X-100 using 0.01 M Tris buffer (pH 7.2) in the presence of 1 mM KCl. Viral lysate was stirred for 45 minutes at room temperature followed by centrifugation at 10,000 × g to get the supernatant containing HA, NA and matrix (M) proteins. The pellet containing NP protein was washed with phosphate buffer saline (PBS), by re-centrifuging at 10,000 × g for 1 hour at 4°C. To remove viral DNA/viral particles the supernatant was centrifuged at 200,000 × g by using Beckman ultracentrifuge L8-80 on 50 Ti rotor (Beckman, USA) for 1 hour to remove the viral DNA and viral particles. The supernatant was collected and dialyzed against 0.01 M PBS for 48 hours. It was again centrifuged at 10,000 × g for 10 minutes to separate M protein out of these preparations and the resulting pellet was suspended in PBS. The supernatant containing HA, NA, polymerase (PM) and non-structural (NS) proteins was collected by centrifuging three times repeatedly at 10,000 × g for 10 minutes at 4°C. The supernatants were dialyzed and the resultant collections were analyzed on 12% polyacrylamide gel. Five bands of AIV proteins separated on the gel were cut, triturated and diluted with 1 ml of normal saline solution (NSS). The material was centrifuged at 1000 × g for 10 min and supernatant was quantified by Lowry's method [21]. Each polypeptide was emulsified with complete Freund's adjuvant and injected @ 4 μg/bird/injection via subcutaneous route in six groups of four birds each (fourth bird was a negative control), twice at two weeks interval, respectively.

### Dot-ELISA

Dot-ELISA was standardized and performed to check the specificity of each polypeptide specific antisera against AI H7N3 virus. Antigen dots were used in different dilutions ranging from Neat virus to 1:4 dilutions with NSS along with a dot containing BSA as a negative control.

### Passive immunization with polypeptides specific antisera

Broiler chicks tested negative for AIV were divided equally into seven group of ten each. These birds were reared under strict isolation and high security conditions in chicken isolators. At the age of two weeks, birds were passively immunized with 4 ml each of the polypeptide specific antisera. Birds were challenged while rearing in chicken isolators at 24 hours post inoculation (PI) with live virus of AI serotype H7N3 at a dose 10^5 ^ELD_50_. The birds were examined for clinical signs, mortality and cloacal shedding, up to 14 days post-challenge (PC).
